# Supplementary material for: Enhancing Production of Medium-Chain-Length Polyhydroxyalkanoates from Pseudomonas sp. SG4502 by tac Enhancer Insertion
Source: Polymers (Basel). 2023 May 12;15(10):2290. doi: 10.3390/polym15102290 (PMC10222556; doi:10.3390/polym15102290)
Supplement: Supplementary file 1 [file polymers-15-02290-s001.zip › polymers-2361315-supplementary.pdf]

Supporting Information

Enhancing Production of Medium-chain-length Polyhydroxyalkanoates from *Pseudomonas* sp. SG4502 by tac Enhancer Insertion

Linxin Song [1,2], Ming Wang [3], Dengbin Yu[1,2], Yu Li [1,4], Hongwen Yu [5] and Xuerong Han [1,3*]

[1] International Cooperation Research Center of China for New Germplasm Breeding of Edible Mushrooms, Jilin Agricultural University, Changchun 130118, China
[2] Jilin Province Key Laboratory of Fungal Phenomics, Jilin Agricultural University, Changchun 130118, China
[3] School of Life Science and Technology Changchun University of Science and Technology Changchun 130022, China
[4] Tianjin Institute of Industrial Biotechnology, Chinese Academy of Sciences, Tianjin 300308, China
[5] Key Laboratory of Wetland Ecology and Environment, State Key Laboratory of Black Soils Conservation and Utilization, Northeast Institute of Geography and Agroecology, Chinese Academy of Sciences, Changchun 130102, China
* Correspondence: hanxuerong@jlau.edu.cn

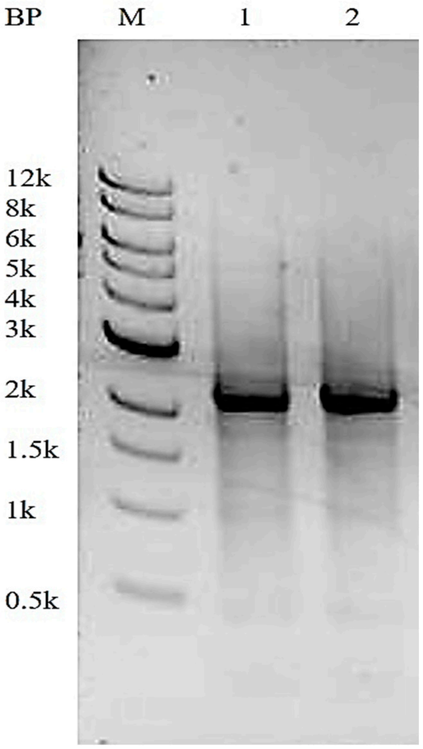

Figure S1. Electrophoretic results of target fragment *C1ZC2* M: DNA Marker GsDL10001, 1,2: PCR product of *C1ZC2* gene.

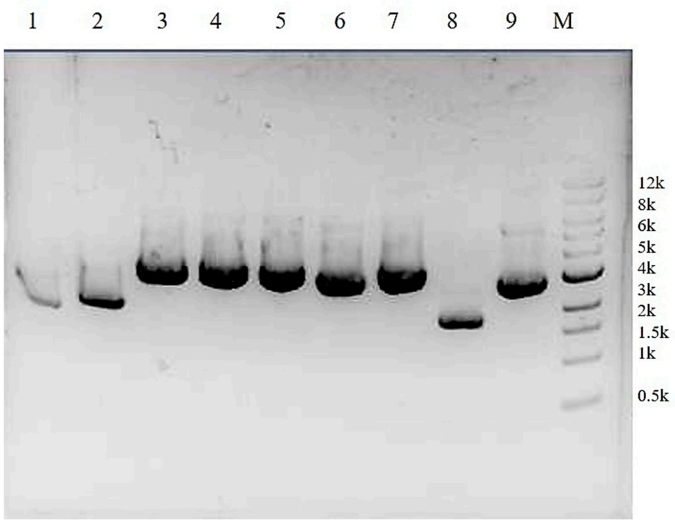

Figure S2. Electrophoretic results of recombinant plasmid pUC19-*C1ZC2*. 1: pUC19 plasmid; 2-9: recombinant plasmid pUC19-*C1ZC2*; M: DNA Marker GsDL10001.

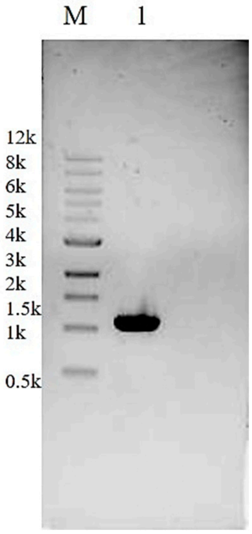

Figure S3. PCR electrophoresis results of *smr* gene. M: DNA Marker GsDL10001; 1: PCR product of *smr* gene.

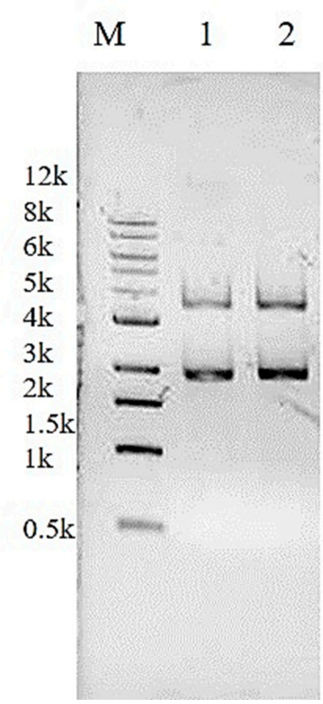

Figure S4. Double enzyme digestion electrophoresis results of recombinant plasmid pUC19-*C1ZC2-smr*. M: DNA Marker GsDL10001; 1,2: Double enzyme digestion products of recombinant plasmid pUC19-*C1ZC2-smr*.

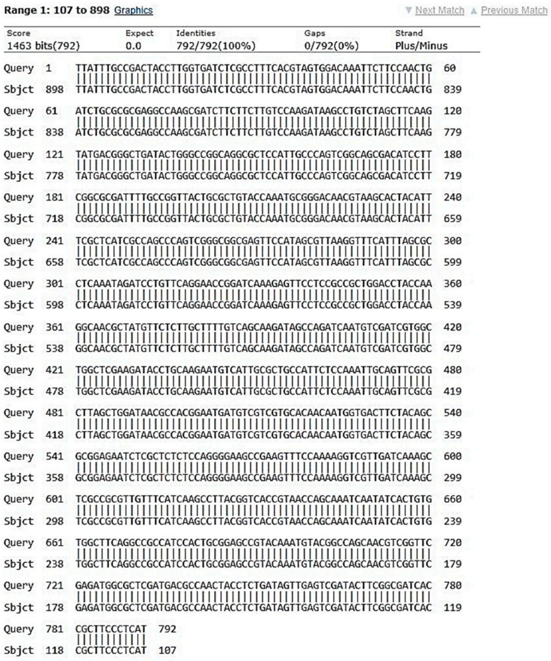

```
Range 1: 107 to 898 Graphics                    ▼ Next Match ▲ Previous Match

Score            Expect  Identities        Gaps          Strand
1463 bits(792)   0.0     792/792(100%)     0/792(0%)     Plus/Minus

Query  1    TTATTTGCCGACTACCTTGGTGATCTCGCCTTTCACGTAGTGGACAAATTCTTCCAACTG  60
Sbjct  898  TTATTTGCCGACTACCTTGGTGATCTCGCCTTTCACGTAGTGGACAAATTCTTCCAACTG  839

Query  61   ATCTGCGCGCGAGGCCAAGCGATCTTCTTCTTGTCCAAGATAAGCCTGTCTAGCTTCAAG  120
Sbjct  838  ATCTGCGCGCGAGGCCAAGCGATCTTCTTCTTGTCCAAGATAAGCCTGTCTAGCTTCAAG  779

Query  121  TATGACGGGCTGATACTGGGCCGGCAGGCGCTCCATTGCCCAGTCGGCAGCGACATCCTT  180
Sbjct  778  TATGACGGGCTGATACTGGGCCGGCAGGCGCTCCATTGCCCAGTCGGCAGCGACATCCTT  719

Query  181  CGGCGCGATTTTGCCGGTTACTGCGCTGTACCAAATGCGGGACAACGTAAGCACTACATT  240
Sbjct  718  CGGCGCGATTTTGCCGGTTACTGCGCTGTACCAAATGCGGGACAACGTAAGCACTACATT  659

Query  241  TCGCTCATCGCCAGCCCAGTCGGGCGGCGAGTTCCATAGCGTTAAGGTTTCATTTAGCGC  300
Sbjct  658  TCGCTCATCGCCAGCCCAGTCGGGCGGCGAGTTCCATAGCGTTAAGGTTTCATTTAGCGC  599

Query  301  CTCAAATAGATCCTGTTCAGGAACCGGATCAAAGAGTTCCTCCGCCGCTGGACCTACCAA  360
Sbjct  598  CTCAAATAGATCCTGTTCAGGAACCGGATCAAAGAGTTCCTCCGCCGCTGGACCTACCAA  539

Query  361  GGCAACGCTATGTTCTCTTGCTTTTGTCAGCAAGATAGCCAGATCAATGTCGATCGTGGC  420
Sbjct  538  GGCAACGCTATGTTCTCTTGCTTTTGTCAGCAAGATAGCCAGATCAATGTCGATCGTGGC  479

Query  421  TGGCTCGAAGATACCTGCAAGAATGTCATTGCGCTGCCATTCTCCAAATTGCAGTTCGCG  480
Sbjct  478  TGGCTCGAAGATACCTGCAAGAATGTCATTGCGCTGCCATTCTCCAAATTGCAGTTCGCG  419

Query  481  CTTAGCTGGATAACGCCACGGAATGATGTCGTCGTGCACAACAATGGTGACTTCTACAGC  540
Sbjct  418  CTTAGCTGGATAACGCCACGGAATGATGTCGTCGTGCACAACAATGGTGACTTCTACAGC  359

Query  541  GCGGAGAATCTCGCTCTCTCCAGGGGAAGCCGAAGTTTCCAAAAGGTCGTTGATCAAAGC  600
Sbjct  358  GCGGAGAATCTCGCTCTCTCCAGGGGAAGCCGAAGTTTCCAAAAGGTCGTTGATCAAAGC  299

Query  601  TCGCCGCGTTGTTTCATCAAGCCTTACGGTCACCGTAACCAGCAAATCAATATCACTGTG  660
Sbjct  298  TCGCCGCGTTGTTTCATCAAGCCTTACGGTCACCGTAACCAGCAAATCAATATCACTGTG  239

Query  661  TGGCTTCAGGCCGCCATCCACTGCGGAGCCGTACAAATGTACGGCCAGCAACGTCGGTTC  720
Sbjct  238  TGGCTTCAGGCCGCCATCCACTGCGGAGCCGTACAAATGTACGGCCAGCAACGTCGGTTC  179

Query  721  GAGATGGCGCTCGATGACGCCAACTACCTCTGATAGTTGAGTCGATACTTCGGCGATCAC  780
Sbjct  178  GAGATGGCGCTCGATGACGCCAACTACCTCTGATAGTTGAGTCGATACTTCGGCGATCAC  119

Query  781  CGCTTCCCTCAT  792
Sbjct  118  CGCTTCCCTCAT  107
```

Figure S5. The sequencing results of recombinant plasmid pUC19-*C1ZC2-smr*. (the accession number of the nucleotide sequences was GenBank: AB448740.1).

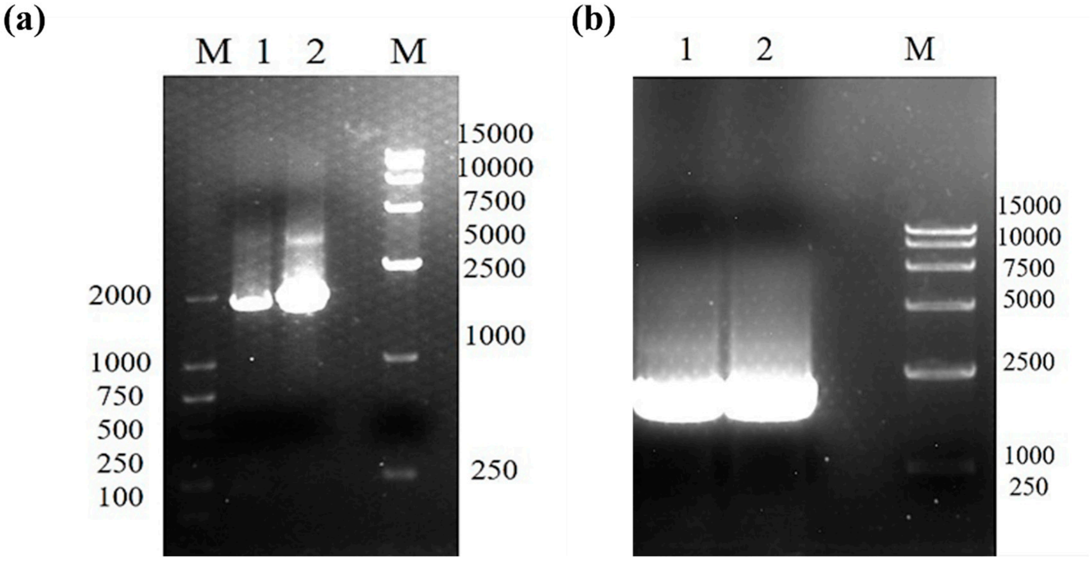

Figure S6. Electrophoretic target genes map. (a) *phaC1,* the left M: DNA Marker DL2000, 1-2: the PCR product of *phaC1,* the right M: DNA Marker DL15000; (b) *phaC2,* 1-2: the PCR product of *phaC2*, M：DNA Marker DL15000.

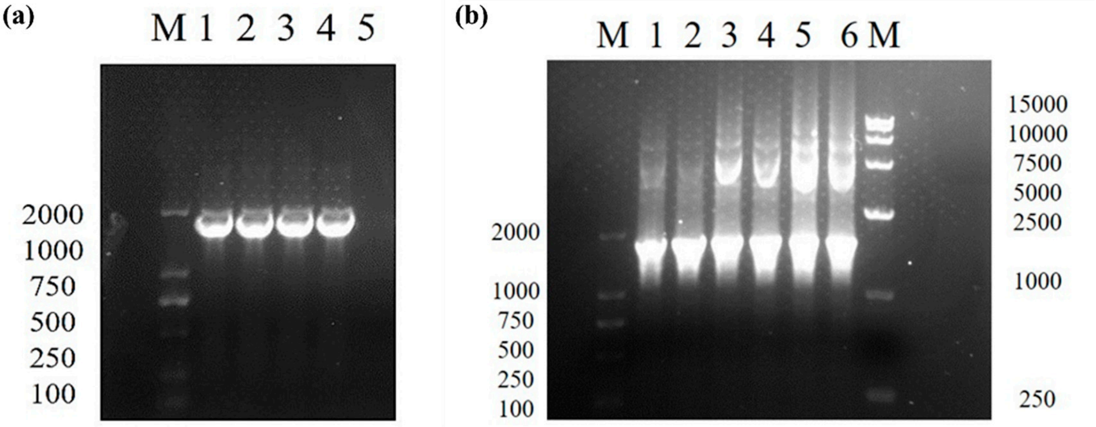

Figure S7. Electrophoresis results of PCR identification. (a) Recombinant plasmid pk18-*phaC1*, M: DNA Marker DL2000, 1: PCR product of *phaC1* gene, 2-4: PCR product of pK18-*phaC1*, 5: PCR product of pK18 empty vector; (b) Recombinant plasmid pk18-*phaC2*, M: DNA Marker DL2000, 1: PCR product of *phaC2* gene, 2-6: PCR product of pK18-*phaC2*, M: DNA Marker DL15000.

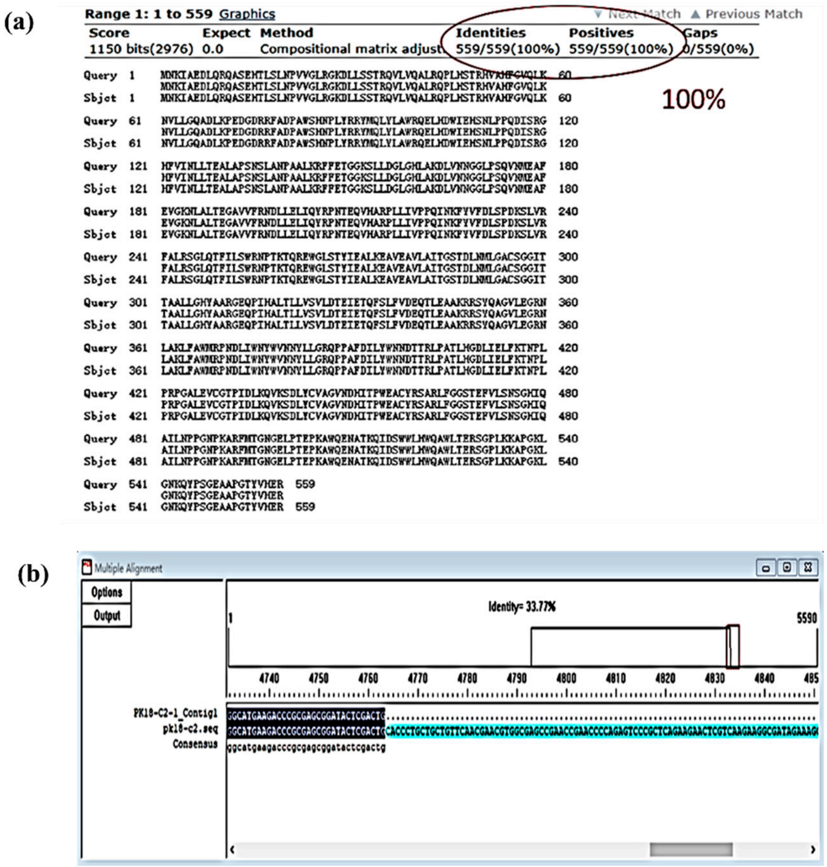

Figure S8. Comparison of sequencing results of recombinant plasmid (a) pK18-*phaC1*, (b) pK18-*phaC2*.

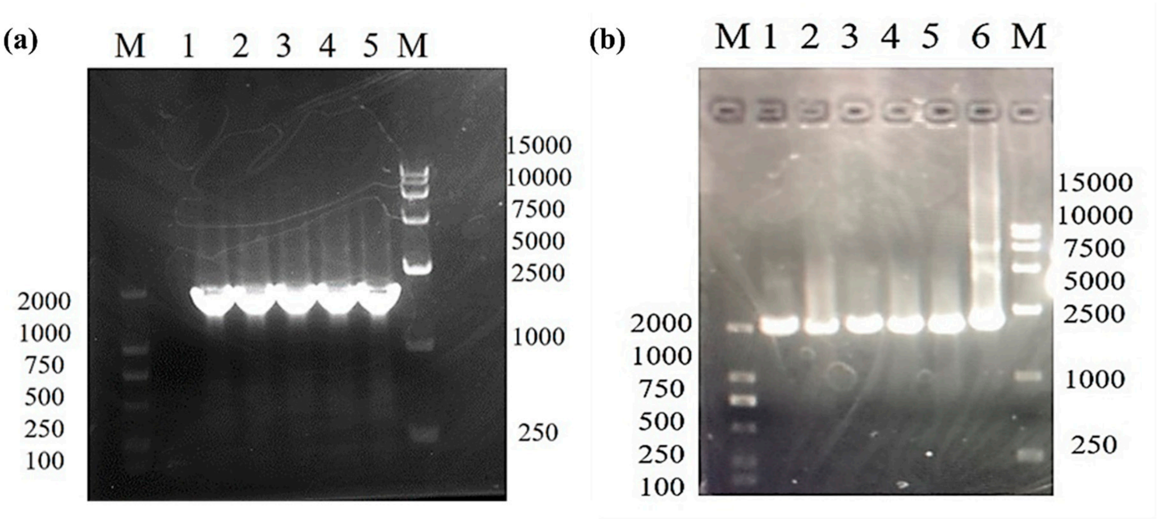

Figure S9. Electrophoresis results of PCR identification. (a) *Pseudomonas* sp. SG4502+ *tac-phaC1*, M: DNA Marker DL2000, 1-5: PCR product of *Pseudomonas* sp. SG4502+*tac-phaC1*, M: DNA Marker DL15000; (b) *Pseudomonas* sp. SG4502+ *tac-phaC2*, M: DNA Marker DL2000, 1-6: PCR product of *Pseudomonas* sp. SG4502+*tac-phaC2*, M: DNA Marker DL15000.

Table S1. Strains and plasmids used in this study.

Strains and plasmids	Related Information	Source
Strains		
E. coli DH5α	Used for the construction of overexpressed plasmids; *F-,φ80dlacZΔM15Δ（lacZYA-arg）U169, deoR, recA1, endA1, hsdR17(rK+, mk+), phoA, supE44, λ-, thi-1, gyrA96, relA1*	This laboratory
E. coli JM109	Construction for knocking out plasmids; *recA1, endA1, gyrA96, thi-1, hidR17, supE44, relA1, Δ (lac-proAB)/F'[traD36,proAB+,lac 1q, lacZΔM15]*	This work
Plasmids		
pK18	Starting plasmid containing strong promoter *tac*, Used as a gene booster; Km^R	This work
pUC19	Starting plasmid, used as gene knockout; Ap^R	This laboratory
pCDFDuet-1	The starting plasmid, which is used to provide the *Smr* gene required for knockout； Sm^R	This laboratory
pK18-*phaC1*	Recombinant plasmids for overexpression of the *phaC1* gene；Km^R	This work
pK18-*phaC2*	Recombinant plasmids for overexpression of the *phaC1* gene；Km^R	This work
pUC19-*C1ZC2*	Recombinant plasmids, knocked out intermediate plasmids containing *the phaC1-phaZ-phaC2* gene； Ap^R； Sm^R	This work
pUC19-*C1ZC2-smr*	Recombinant plasmid, knockout intermediate plasmid containing *the phaC1-phaZR-smr-phaZF-phaC2* gene； Ap^R； Sm^R	This work

Table S2. Primers used in this study.

Primers	Description	Source
phaC1-F	CGGATCCCCGGGTACCGGGACAACGGAGCGTCGTAG	This work
phaC1-R	ACGAATTCGAGCTCGGCGGAACACGAAGGGGCTGGG	This work
phaC2-F	CTTGGTCGGTCATTTGGCAATCTGCAGCAGGCAGTC	This work
phaC2-R	GGACTCTGGGGTTCGGTTCGGCTCGCCACGTTCGTT	This work
tac-phaC1-F	GATAAGCCCGGATCCCCGGGTACCGGGACA	This work
tac-phaC1-R	ACGAATTCGAGCTCGGCGGAACACGAAGGGGCTGGG	This work
tac-phaC2-F	CGGTCATTTGGCAATCTGCAGCAGG	This work
tac-phaC2-R	TCGCTCTCGTCGATCCACT	This work
q-phaC1-F	TGTTCCGCAACGACCTGCTA	This work
q-phaC1-R	GAATCGCACCAGGCTCTTGTCC	This work
q-phaC2-F	CGTCCGTTTCGCCGATCCCA	This work
q-phaC2-R	TCGCTCTCGTCGATCCACT	This work
q-phaZ-F	CCTTCGTGTTCCGCACCAT	This work
q-phaZ-R	ACGTCGAAGGCGATCACCTC	This work
pC1ZC2-F	GACGGCCAGTGAATTGGGAACAACGACACCACGCGCCTGCC	This work
pC1ZC2-R	TGATTACGCCAAGCTAGCAGGTCGTCGATCAGGTGGCGCAG	This work
Smr-F	GACCCTGCGCACCGCGACATAAGCGGCTATTTAACGACCC	This work
Smr-R	TGGAGGTAGCGCCGCGCCGCAGTCTCACGCCCGGAGCGTA	This work
